# Who is Exposed to HIV Prevention Interventions? An Assessment of Associated Factors Among Adolescent Girls and Young Women in South Africa

**DOI:** 10.1007/s10461-023-04023-1

**Published:** 2023-03-01

**Authors:** Kim Jonas, Daniel Beattie, Rik Crutzen, Catherine Mathews

**Affiliations:** 1 Health Systems Research Unit, South African Medical Research Council, Francie Van Zijl Drive, Parowvallei, Tygerberg, PO Box 19070, Cape Town, 7505 South Africa; 2 Adolescent Health Research Unit, Division of Child and Adolescent Psychiatry, Faculty of Health Sciences, University of Cape Town, Cape Town, South Africa; 3 Department of Health Promotion, CAPHRI, Faculty of Health, Medicine and Life Sciences, Maastricht University, Maastricht, The Netherlands

**Keywords:** HIV, HIV prevention interventions, Sexual risk factors, South Africa

## Abstract

This study examined the prevalence of HIV risk factors and their association with intervention exposure among adolescent girls and young women (AGYW) living in six South African districts in which a combination HIV-prevention intervention was being implemented. A cross-sectional household survey was conducted from 2017 to 2018 among a representative sample of AGYW aged 15–24 years living in the six districts. We used an electronic questionnaire for self-reported demographic and behavioural questions and blood samples were taken to confirm HIV status in the laboratory. Chi-Squared tests and multivariate binary logistic regression were used to examine associations between demographic characteristics, HIV acquisition and transmission risk factors and the likelihood of participating in any of the key components of the combination HIV-prevention intervention. Among the 4399 participants, 45.3% reported inconsistent condom use with casual partner and 46.6% with a main partner. Almost half of participants (47.8%) had participated in one or more components of the HIV-prevention intervention, and in a multivariate logistic regression, those reporting a higher number of HIV risk behaviours were no more (or less) likely to participate. Participants who were not in high school were significantly less likely to have participated in the intervention compared to those still in high school, when adjusting for age and HIV risk factors. The barriers to access and uptake of combination HIV prevention interventions among AGYW who are out of the education system need to be explored and combination HIV prevention interventions and implementation strategies need to be tailored to reach this population.

## Background

HIV/AIDS presents a major global health challenge with approximately 37.9 million people living with the disease [1]. It is one of the leading causes of morbidity and mortality worldwide [2]. Sub-Saharan Africa is disproportionately affected accounting for more than half (65%) of the global burden and in particular, around 26% of people living with HIV are estimated to live in South Africa [3]. Whilst the number of new infections is reducing in South Africa [4], incidence remains the highest worldwide with 23% of new HIV infections occurring in the country [3]. AIDS is responsible for a significant burden of premature mortality and is the second leading cause of death in people aged 15–24 years old in South Africa [5]. In addition to physical health consequences, infection can lead to wider social consequences. It is associated with a variety of psychological health problems such as depression and anxiety as a result of the diagnosis or through stigma and social rejection related to the disease [6]. Whilst poor education is a risk factor for HIV [7], infection can also result in poorer educational attainment [8]. Additionally, it has a negative economic impact on households [9] and both educational attainment and economic status indirectly contribute to poorer health outcomes in adulthood [10].

Whilst some infections may be acquired perinatally, the primary mode of transmission is through heterosexual intercourse in sub-Saharan Africa [11]. Inconsistent condom use, multiple sexual partners, age-disparity over 5 years between partners, early sexual debut and rape are all well documented risk factors for HIV acquisition and transmission [12–14]. Additionally, alcohol and substance use has been demonstrated to increase sexual risk behaviour such as multiple sexual partners and inconsistent use of protection [15, 16]. Previous pregnancy during adolescence has also been associated with increased rates of HIV infection as these may share similar risk factors [17]. Previous studies have demonstrated that poor access and inadequate provision of sexual and reproductive health services (SRH), and lack of sexual knowledge are key drivers of sexual risk behaviours in South Africa [18]. School enrolment and higher levels of education are protective factors against HIV acquisition [7, 12]. School attendance is associated with lower sexual risk behaviours and contributes to reducing the spread of sexually transmitted infections [19]. Education has been shown to be an effective strategy in improving sexual health literacy across various settings [20] and this has been proven to increase the use of contraceptives, including condom use, and reduce risky sexual behaviour [21].

Adolescent girls and young women (AGYW) bear a disproportionate burden of HIV infection with 30% of new infections occurring in those aged 15–24 years [22]. During adolescence, behavioural changes occur leading to sexual debut, concurrent sexual partners and inconsistent use of protection, all of which result in increased risk of acquisition during this period [23]. Almost two thirds of new infections in adolescents occurred in Sub-Saharan Africa reflecting on the social, cultural and economic determinants of HIV in this region [24]. Gender-power disparities and unequal power in relationships lead to AGYW being vulnerable to having multiple sexual partners, high age-disparities with partners, forced sexual relations and low ability to negotiate for condom use [25, 26]. Furthermore, socioeconomic circumstances have resulted in high levels of ‘transactional sex’; in which girls have sex, often with older men, on the premise of financial reward. These relationships usually come with unequal gender power dynamics [27]. Consequentially in South Africa, AGYW have a threefold higher prevalence than their male peers [11]. Reaching AGYW with HIV prevention and treatment interventions is key to controlling the HIV epidemic in this setting.

**Fig. 1:**
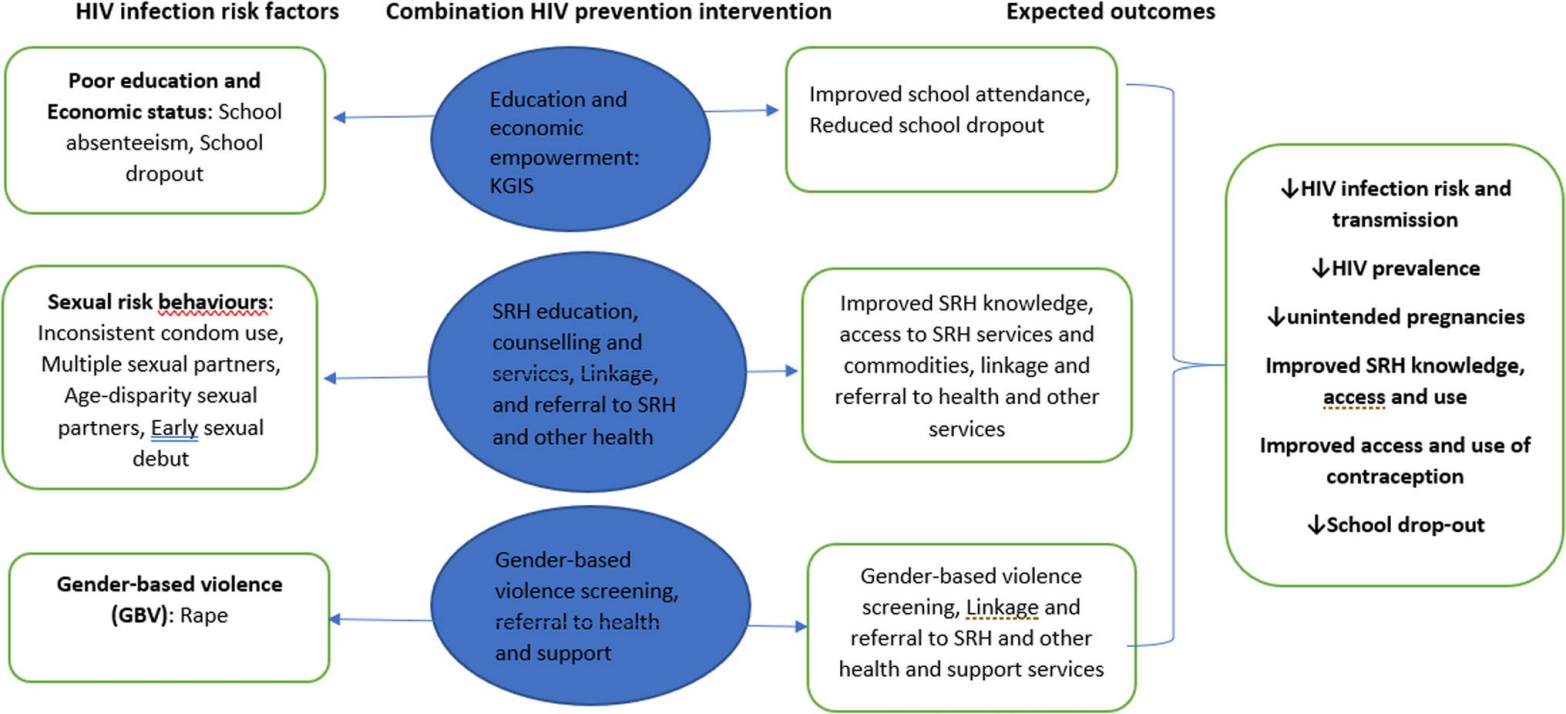
Conceptual framework of the combination HIV prevention intervention to reduce HIV transmission among AGYW. *KGIS* Keeping Girls In School, *SRH* Sexual and Reproductive Health, ↓ reduce

Tackling HIV is a key priority at national level, reflected in the South African National Strategic Plan for HIV, TB and STIs (NSP)-which targets a reduction in new infections with specific focus on priority populations, such as the AGYW subgroup population [28]. Aligned to the NSP; a combination HIV-prevention intervention was developed and implemented in ten high burden districts in South Africa between 2016 and 2019. This comprised of several different components which aimed to reduce new HIV acquisition and onward transmission of current infection by targeting key risk factors such as school dropout, poor sexual health literacy, poor access to sexual and reproductive health services and lack of societal empowerment. The main intervention components were-Soul Buddyz, Rise and Women of Worth Clubs (hereby referred to as ‘Clubs’) and the Keeping Girls in School programme-which specifically targeted young women and girls. The Keeping Girls In School (KGIS) programme identified and targeted AGYW at risk of dropping out of high school and provided support to keep them enrolled (such as homework support clubs) as well as sexual and reproductive education and referral to SRH services. The Clubs targeted AGYW aged 15–19 years (in school) and 19–24 years (out of school) offering activities for female empowerment in response to the gender-based risk factors which have led to AGYW being particularly vulnerable to sexual and reproductive health problems, and provided sexual and reproductive education and referral to services. Further details about the intervention components are detailed in Mathews et al. [29]. Figure 1 shows a conceptual framework of how the combination HIV prevention intervention aimed to address the HIV risk factors among AGYW.

For interventions to have a maximal effect in reducing HIV incidence, they must reach the population most at risk of acquiring and/or transmitting the disease [30]. Assessments of existing interventions have shown persistently poor levels of service uptake amongst those with high HIV incidence and this highlights the need for tailoring interventions to cater for the specific needs of their target population [31]. To do such, it is necessary to fully understand the factors which may influence intervention exposure. Thus, the purpose of this study was to assess the prevalence of risk factors for HIV acquisition and onward transmission, and their association with exposure to the key components of the combination HIV-prevention interventions, amongst AGYW living in six of the ten intervention districts in South Africa. We aimed to provide insight into the risk-profile of the population and investigate whether AGYW at highest risk of HIV acquisition and/or transmission were more likely to have participated in components of the intervention.

## Methods

### Study Design and Population

This study was designed in adherence with STROBE guidelines for reporting observational studies [31]. We performed a secondary data analysis on the HERStory Study conducted during 2017–2018 [29]. This was a cross-sectional survey in which we randomly sampled 21,193 households in 6 high HIV burden districts where HIV-prevention interventions were implemented, with the aim of recruiting 7300 AGYW from these households. The districts were: City of Cape Town (Western Cape), Ehlanzeni (Mpumalanga), OR Tambo (Eastern Cape), City of Tshwane (Gauteng Province), King Cetshwayo (KwaZulu-Natal), and Zululand (KwaZulu-Natal). Representative census small area layers were chosen from each district and a systematic random sample of 35% of available houses in these areas were selected. All adolescent girls and young women aged 15–24 years living in the sampled houses were eligible for this study. The survey consisted of a variety of domains including demographic data, health and well-being, sexual experiences, access to healthcare and interventions, sexual health knowledge, gender power imbalances, and pregnancy, STI and HIV history. Data were self-reported by participants in an interviewer-administered, electronic questionnaire on a tablet. To eliminate response bias, for questions covering stigmatised or sensitive topics including questions related to sexuality, the participant was invited to self-complete the questionnaire privately without the fieldworker viewing the response. All participants who completed this survey were included in this study. Detailed description of the study design can be found in Mathews et al. [29].

### Measures

Data was collected from participant’s responses to the 2017–2018 HERStory Study. In total, 4426 participants from across the 6 districts participated in the study. There were 27 participants who fell outside the 15–24-year-old age range and so were excluded from analysis. This resulted in 4399 eligible responses which were available for analysis, a sample realization of 60.6%. Participant’s age, district, level of education, and school absenteeism were recorded. Level of education was assessed by highest grade completed and whether the participant had dropped out of high school without completing Grade 12. Dropping out of school without completing grade 12 was used as a proxy indicator of poor education. Further analyses involve demographic factors and other risk factors associated with intervention participation are published elsewhere [32–34].

#### Participation in the Interventions

The dependent variable was a participant’s exposure to any of several components of the combination HIV-prevention intervention. Intervention exposure was defined as participation in Soul Buddyz Clubs, past or present enrolment in Rise Clubs, Women of Worth Programme, homework support programme, having ever attended a KGIS programme or received a cash incentive from the Women Worth Programme. We composed a composite indicator of participation in above intervention components, where AGYW were considered as ‘exposed’ to the intervention if they have answered ‘yes’ to any one of the questions assessing their participation in the aforementioned components. Participants who answered ‘no’ to all of these were considered non-exposed to interventions.

#### Sexual Experiences and Behaviours (HIV Risk Factors)

To measure HIV risk factors for acquisition and transmission, we included the following nine variables: (1) early sexual debut (before 15 years of age); (2) inconsistent condom use with main partners in the past 3 months; (3) inconsistent condom use with casual partners in the past 3 months (reporting not always using a condom); (4) multiple (≥ 2) sexual partners in past 3 months; (5) a sexual partner ≥ 5 years older than the participant in the past year; (6) having had an sexual partner living with HIV in the past year; (7) ever had transactional sex; (8) ever been forced or threatened into having sex; and (9) ever had sex under the influence of alcohol or drugs.

To compile risk factors into an overall composite assessment of AGYW’s HIV acquisition or transmission risk, a scale index of HIV risk was constructed based on a modified version of the HIV risk index created by Sales and Sheth [35]. We scored participants with 1 point for each of the nine risk factors they reported to give an overall number of risk indicators per person, which could range from a score of 0 to 9, with a higher score indicating a higher risk of HIV acquisition or transmission. Although there may be differences in the effect of each risk factor on HIV transmissibility, this provided a comprehensive overall measure of HIV risk behaviour and thus highlights AGYW with multiple risk factors who would be important targets for intervention exposure.

#### Knowledge of HIV Transmission

We used four questions on common misconceptions to measure knowledge of HIV transmission as a possible risk factor. Participants were asked if they agreed or disagreed to these items:

A person can get HIV or AIDS because of witchcraft

A guy who has HIV or AIDS can be cured by having sex with a girl who is a virgin

If a guy is circumcised there is no chance he could get HIV

If you have anal sex with someone who is living with HIV, you can become infected with HIV

An HIV knowledge index was created to assess the number of inaccurate beliefs held by each participant. For each of the above statements, holding the inaccurate belief was scored as 1 and the correct belief was scored 0, resulting in a scale of between 0 and 4 possible inaccurate beliefs per person. Thus, a higher score was associated with poorer HIV knowledge.

#### HIV Testing and HIV Status

To ascertain access to HIV testing services, participants were asked whether they had ever had an HIV test. All participants were asked to give a blood sample which was tested for HIV using two 4th Generation ELISA screening tests. Samples which were positive for HIV following both screening tests were given a confirmatory test using the Western Blot technique. AGYW who were seropositive on both screening tests and the Western Blot were classified as living with HIV. Those who were seronegative after ELISA screening tests were classified as not living with HIV. AGYW with discrepant results between screening and confirmatory tests were given further laboratory testing to ascertain HIV status. The list of variables used for this study can be found in Appendix.

### Data Analysis

Of the 4399 responses analysed, 1390 AGYW had never had sex. For analysis, it was assumed and coded that these AGYW displayed none of the risk factors investigated. For many of the questions in the study, participants had the option of ‘Prefer not to say’. These responses were recorded in the descriptive section of the results. However, for the logistic regression analyses participants who responded with ‘prefer not to say’ were excluded.

Data was analysed using IBM Statistical Package for the Social Sciences (SPSS) Statistics 25 and STATA 17. All variables were coded (see Appendix) and then descriptive statistics including frequencies were performed to describe the sample, including demographic characteristics, prevalence of HIV risk factors and intervention exposure. After this, data were assigned frequency weighting to account for district and census small area layer size and for disparities in response rate. Districts and census small area layers with higher populations were assigned higher weighting than those with smaller populations to ensure a sample representative of the population under investigation. To measure intervention exposure, we included questions about the components of the intervention that were branded and/or easy to identify for the AGYW. We also showed the participant the intervention logos where appropriate, or the health education materials associated with that intervention component, to ensure easy identification. For example, the “Keeping Girls at School” programme was not necessarily known by KGIS among the AGYW who participated in it. Instead, we asked whether the participant had attended a health education session at school in which a set of flipcharts was used, and we showed a picture of the flipcharts. Then AGYW who were enrolled in the KGIS programme were able to recognize the intervention material that way.

Pearson’s Chi Square tests were performed to assess the factors associated with intervention exposure. Simple logistic regression was performed to measure the association between the risk behaviour index and intervention exposure, and the knowledge index and intervention exposure. Finally, a multivariate logistic regression model was performed to examine whether the HIV risk index and the knowledge of HIV transmission index were associated with intervention exposure, adjusting for covariates district, age, and school enrolment/dropout. Multicollinearity statistics were conducted on the variables to be included in the regression model and none had a variance inflation factor (VIF) of more than 5 indicating that collinearity assumptions were not violated. A significance level of p < 0.05 was used for all analyses.

## Results

### Demographic Characteristics

The responses of 4399 AGYW between the ages of 15–24 years were analysed, giving a response rate of 60.6%. The mean age of respondents was 19.1 years with standard deviation (SD) of 2.7. Table 1 describes the demographic characteristics of the participants. Respondents aged 15–19 years made up 2515 (57.2%) of the sample whilst 1884 (42.8%) were aged 20–24 years. Slightly more than half of the sample were currently enrolled in school (2518, 57.2%), while 1174 (26.7%) had completed Grade 12 and 707 (16.1%) had dropped out of school without completing grade 12. Of those currently enrolled in school, 100 (4.0%) had been absent from school more than 1 day a week, and considered as regularly absent.

**Table 1:** Socio-demographic characteristics and intervention exposure of the AGYW included in the HERStory Study, 2018–19 (N = 4399)

Variable	Total prevalence		Exposed to interventions		Association with intervention exposure	
	N	%	N	%	χ^2^	p-value
Total population	4399	100	2103/4399	47.8	–	–
Demographics						
Age (years)						
15–19	2515	57.2	1364/2515	54.2	97.3	0.00*
20–24	1884	42.8	739/1884	39.2	–	–
District						
City of Cape Town	377	8.6	149/377	39.5	177.9	0.00*
City of Tshwane	767	17.4	360/767	46.9	–	–
Ehlanzeni	803	18.3	481/803	59.9	–	–
King Cetshwayo	748	17.0	259/748	34.6	–	–
OR Tambo	690	15.7	426/690	61.7	–	–
Zululand	1014	23.1	428/1014	42.2	–	–
Education						
In school	2518	57.2	1455/2518	57.8	243.8	0.00*
Completed year 12^*^	1174	26.7	437/1174	37.2	–	–
Dropped out before Year 12	707	16.1	211/707	29.8	–	–
Absenteeism^a^						
Not regularly absent	2418	96.0	1395/2418	57.7	0.21	0.65
Regularly absent	100	4.0	60/100	60.0	–	–
HIV status						
HIV status						
Living with HIV	568	12.9	234/568	41.2	11.4	**0.001**
Not living with HIV	3831	87.1	1869/3931	48.8	–	–
Accessed HIV Testing						
Never	968	22.0	489/968	50.5	3.65	0.06
Had accessed	3431	78.0	1614/3431	47.0	–	–
Sexual Behaviour and Experiences						
Ever had sex						
Yes	3009	68.4	1382/3009	45.9	13.5	**0.00**
No	1390	31.6	721/1390	51.9	–	–
Sexual Debut^b^						
Early (≤ 14 Years)	259	5.6	124/259	47.9	14.06	**0.01**
Not early (≥ 15 years)	2746	62.4	1257/2746	45.8	–	–
Never had sex/prefer not to say	1394	31.7	724/1394	34.4		
Multiple Sexual Partners in past 3 months						
≥ 2 partners	570	13.0	284/570	49.8	0.67	0.40
≤ 1 partners or never had sex	3623	82.4	1737/3623	47.9	–	–
Prefer not to say	206	4.7	–	–	–	–
Condom use with main partner in past 3 months						
Inconsistent	2049	46.6	908/2049	44.3	20.7	**0.00**
Consistent/did not have sex	2157	49.0	1107/2157	51.3	–	–
Prefer not to say	193	4.4	–	–	–	–
Condom use with casual partner in past 3 months						
Inconsistent	1994	45.3	872/1994	43.7	23.1	**0.00**
Consistent/did not have sex	2019	45.9	1036/2019	51.3	–	–
Prefer not to say	386	8.8	–	–	–	–
Age of Sexual Partners in past in the past year						
≥ 5 years older	1022	23.2	424/1022	41.5	21.3	**0.00**
< 5 years difference or no partners	3377	76.8	1679/3377	49.7	–	–
Sexual Partner HIV Status past in the past year						
Had partner living with HIV	137	3.1	53/137	38.7	4.85	**0.03**
All partners were NOT living with HIV or did not have partner	4196	95.4	2024/4196	48.2	–	–
Prefer not to say	66	1.5	–	–	–	–
Ever had transactional sex						
Yes	424	9.6	221/424	52.1	3.50	0.06
No	3975	90.4	1882/3975	47.3	–	–
Ever had sex after being forced or threatened?						
Yes	143	3.3	80/143	55.9	4.04	**0.04**
No	4158	94.5	1971/4158	47.4	–	–
Prefer not to say	98	2.2	–	–	–	–
Ever done something sexual following drug or alcohol use?						
Yes	195	4.4	87/195	44.6	0.83	0.36
No	4204	95.6	2016/4202	48.0	–	–
HIV Knowledge and Beliefs						
Having sex with a virgin can cure HIV						
Agree	346	7.9	179/346	51.7	2.32	0.13
Disagree	4053	92.1	1924/4053	47.5	–	–
Circumcised males cannot contract HIV						
Agree	853	19.4	428/853	50.2	2.38	0.12
Disagree	3546	80.6	1675/3546	47.2	–	–
Witchcraft is a cause of HIV						
Agree	263	6.0	130/263	49.4	0.30	0.59
Disagree	4163	94.0	1973/4163	47.7	–	–
Cannot contract HIV via anal sex						
Agree	1550	35.2	718/1550	46.3	2.11	0.15
Disagree	2849	64.8	1385/2849	48.6	–	–
ARV cannot prevent transmission of HIV from a pregnant lady to her baby						
Agree	1448	32.9	665/1448	45.9	3.06	0.08
Disagree	2951	67.1	1438/2951	48.7	–	–

Bold: Indicates association with likelihood of being exposed to interventions at p < 0.05 level

*HIV*  human immunodeficiency virus, *ART* antiretroviral treatment

^a^For school absenteeism, only those who reported being currently enrolled in school were asked this. Hence N = 2518

^b^For this question, only those who had previously had sex were asked. Hence N = 3009

*Also referred to as Grade 12

The percentage of the population in the weighted sample who were living with HIV was 12.9%. Across districts, participants in the City of Cape Town had the lowest HIV prevalence (3.4%) whilst Zululand participants had the highest prevalence (15.5%) (disaggregated data not shown). HIV prevalence ranged by age group, with 185 (7.4%) of 15–19 years olds living with HIV whilst amongst 20–24 years old, 388 (20.3%) were living with HIV. HIV prevalence amongst those currently enrolled in school was 214 (8.5%), the prevalence in those who had completed Grade 12 was 158 (13.5%) and 196 (27.7%) in those who had dropped out of school.

Of the participants surveyed, 2103 (47.8%) had been exposed to components of the HIV-prevention intervention. Intervention exposure ranged across the 6 districts ranged from 259 (34.6%) in King Cetshwayo, to 426 (61.7%) in the OR Tambo district. Over half (1364, 54.2%) of 15–19 years olds had been exposed to the intervention, however, among the 20–24 years olds only 739 (39.2%) had. Of those currently enrolled in school, 1455 (57.8%) had been exposed to the intervention. In comparison, 437 (37.2%) of those who had completed grade 12 and 211 (29.8%) of those who had dropped out of school before Grade 12 had been exposed to the intervention. Almost half (1869; 48.8%) of the participant not living with HIV had been exposed to the intervention, compared with 41.2% of those who were living with HIV.

### Prevalence of Risk Factors and Association with Intervention Exposure

Participants reported relatively high prevalence of HIV risk behaviours (Table 1) including early sexual debut (5.6%); multiple sexual partners (13.0%); inconsistent condom use with casual partner (45.3%) and main partner (46.6%); sexual partner who was 5 or more years older (23.2%); and transactional sex (9.6%). They also reported other factors that rendered them more vulnerable to HIV including being coerced into sex (3.3%) and “doing something sexual” following using drugs or alcohol (4.4%).

Participants’ scores on the HIV transmission risk index ranged from 0 to 9, with 35.7% of participants reporting no risk factors and 25.6% reporting two risk factors (Table 2). Substantially fewer of those in the 15 to 19 year age group (48.3%) reported one or more HIV acquisition or transmission risk factors, compared with 85.8% of the participants in the 20 to 24 year age group. Participant’s scores on the knowledge index ranged from 0 to 4, with almost half of participants (46.5%) endorsing none of the inaccurate beliefs, and 41.2% endorsing one inaccurate belief (Table 2).

**Table 2:** Distribution of knowledge and HIV transmission risk scores of AGYW included in the HERStory Study, 2018–19 (N = 4399)

Variable	Risk scores 15–19 year old	Risk scores 20–24 year old	Risk scores Total	
	N (%)	N (%)	N	(%)
Knowledge Index scores (n = 4399)				
0	1086 (43.1)	958 (50.9)	2044	(46.5)
1	1090 (43.2)	724 (38.5)	1814	(41.2)
2	284 (11.3)	155 (8.2)	439	(10.0)
3	50 (1.9)	38 (2.0)	88	(2.0)
4	10 (0.4)	4 (0.2)	14	(0.3)
HIV Transmission Risk Index scores (n = 4399)				
0	1303 (51.7)	268 (14.2)	1570	(35.7)
1	268 (10.6)	257 (13.7)	525	(11.9)
2	506 (20.1)	621 (33.0)	1127	(25.6)
3	270 (10.7)	446 (23.7)	716	(16.3)
4	124 (4.9)	199 (10.6)	323	(7.3)
5	37 (1.5)	57 (3.0)	94	(2.1)
6	9 (0.4)	22 (1.2)	31	(0.7)
7	3 (0.1)	9 (0.5)	12	(0.3)
8	0	0	0	0
9	0	1 (0.1)	1	(0.02)

Table 3 shows multivariate logistic regression model. When other factors were kept constant, age did not have a significant effect on the likelihood of being exposed to interventions. However, compared with participants who were still in school, AGYW who had completed Grade 12 were significantly less likely to have been exposed to interventions (AOR 0.45, 95% CI 0.40–0.52, p < 0.001), as were those who had dropped out before Grade 12 (AOR 0.33, 95% CI 0.27–0.39, p < 0.001) when adjusting for all other variables in the model (Table 3). Compared to the City of Cape Town, participants from City of Tshwane (AOR 1.30, 95% CI 1.04–1.60, p = 0.01), Ehlanzeni (AOR 2.19, 95% CI 1.73–2.77, p < 0.001) and OR Tambo (AOR 2.23, 95% CI 1.82–2.73, p < 0.001) all had greater likelihood of intervention exposure. King Cetshwayo had a lower likelihood of intervention exposure when compared to City of Cape Town (AOR 0.71, 95% CI 0.56–0.89, p < 0.01) (Table 3). When adjusting for other factors in the model, there was no significant association between the behavioural risk index and intervention exposure (AOR 1.08, 95% CI 0.98–1.05, p = 0.34) or the knowledge index and intervention exposure (AOR 1.20, 95% CI 0.94–1.07, p = 0.03).

**Table 3:** Factors associated with exposure to AGYW programme interventions, HERStory Study, 2018–19: results of multivariate logistic regression

Variable	N	Crude Odds Ratio	95% CIs	Adjusted Odds Ratio	95% CIs
Age (years)					
15–19 (Ref)	2515	**–**	**–**	**–**	
20–24	1884	**0.56**	**0.51–0.63**	0.91	0.80–1.05
District					
Cape Town (ref)	377	**–**	**–**	**–**	
City of Tshwane*	**767**	**1.35**	**1.09–1.68**	**1.30**	**1.04–1.60**
Ehlanzeni**	**803**	**2.37**	**1.89–2.97**	**2.19**	**1.73–2.77**
OR Tambo**	**690**	**2.48**	**2.03–3.02**	**2.23**	**1.82–2.73**
King Cetshwayo*	748	0.81	0.65–1.01	**0.71**	**0.56–0.89**
Zululand	1014	1.12	0.90–1.39	0.98	0.78–1.22
Education					
In School (ref)	2518	**–**	**–**	**–**	
Completed Gr 12**	**1174**	**0.43**	**0.38–0.48**	**0.45**	**0.40–0.52**
Dropped out before Gr 12**	**707**	**0.33**	**0.28–0.39**	**0.33**	**0.27–0.39**
Behavioural Risk Index	**–**	**0.93**	**0.91–0.97**	1.08	0.98–1.05
Knowledge Risk Index*	**–**	1.05	0.97–1.12	**1.20**	**1.02–1.41**

Bold: Significant, * p ≤ 0.05, ** p < 0.001 l

## Discussion

This study documents the prevalence of key risk factors for HIV acquisition and/or transmission amongst a representative sample of 15–24 years olds from 6 high burden districts in South Africa in which a combination HIV prevention intervention was being implemented. We found a high prevalence of inconsistent condom use with main and casual partners, with almost half of the participants reporting each of these risk factors. This is aligned with previous research suggesting that condom use amongst those aged 15–24 years in South Africa is decreasing [36, 37] and although condom use at last sex is high, consistent condom use is considerably lower [38]. Of all sexual risk behaviours, unprotected sexual intercourse has the greatest risk of HIV acquisition or transmission [39].

Correct and consistent condom use has been shown to reduce the risk of HIV transmission and promoting condom use has been identified as a key strategy in reducing HIV/AIDS in South Africa [40, 41]. There is evidence that strategies which promote condom use and HIV education are successful in reducing HIV-risk behaviours [42]. Appropriately, promoting condom use was a key strategy of the combination HIV prevention intervention that is the subject of this study, and this programme was thus addressing a key need in the population of AGYW in the intervention districts.

We created a HIV risk index comprised of nine risk behaviours, and we found that 64.3% of participants reported one or more of these behaviours with substantially more of older participants reporting such behaviours. This is further evidence that that intervention was being implemented in districts in which AGYW (especially those in the 20 to 24 year age group) were at high risk of HIV acquisition or transmission.

Slightly over half of the participants held one or more inaccurate beliefs about the transmission of HIV, For example, over one third falsely believed that it was impossible to contract HIV through anal sex, despite evidence suggesting that unprotected anal sex had a higher per act transmission rate than penile-vaginal intercourse [36]. Previous research from various contexts has suggested that misconceptions surrounding HIV transmission are linked with high levels of risk behaviour in young people including unprotected intercourse [39, 43].

Almost half of participants had been exposed to one or more components of the combination HIV prevention programme that was being implemented in the district in which they lived. When controlling for demographic characteristics (age, schooling enrolment, district), there was no association between having a higher number of risk factors and participation in the intervention. Likewise, there was no association between a higher number of incorrect beliefs about HIV transmission and intervention exposure. This indicates that the combination HIV prevention intervention was no more (or less) likely to reach AGYW at highest risk for HIV.

In the multivariate model, we found an association between being in the education system and intervention exposure, with those enrolled in school being more likely to report intervention participation compared with those who completed schooling or dropped out before completion. School dropout and poor educational attainment is associated with a higher level of sexual risk behaviours and increased likelihood of having HIV and thus this group can be considered as a high-risk group [19, 44], and it is important to tailor strategies to reach this group with combination HIV prevention interventions. We found that high school absentee rates did not alter the likelihood of intervention exposure among participants still in school. There is evidence that school absenteeism is associated with higher incidence of HIV [45], and the intervention that is the subject of this study was no more (or less) successful at reaching this group of AGYW.

Interventions which encourage remaining in school and promoting educational attainment targeted towards those currently enrolled have positive effects in reducing sexual risk behaviours and HIV prevalence [46]. Increasing educational attainment has continuously been emphasised as a strategy for achieving gender equality and reducing HIV risk among AGYW in South Africa [47]. However, given the extremely high school dropout rate in South Africa, where around 60% of first graders were expected to drop out before grade 12 [48], it is vital to ensure that interventions do not neglect this important group. There are challenges to reaching AGYW who are no longer in school including structural barriers such as identifying appropriate places and time to reach considering their job and family commitments, and the cost of reaching them [30].

In the multivariate model, intervention exposure was highest among AGYW in OR Tambo and lowest in King Cetshwayo, City of Cape Town and Zululand. King Cetshwayo and Zululand had the highest HIV prevalence among AGYW, which is not surprising given these districts are located within KwaZulu-Natal province; the epicentre of the HIV epidemic in South Africa [37]. It could be argued that maximal effect would be achieved through the prioritisation of these districts over those areas with a lower HIV prevalence. However, extreme geographical prioritisation of resources may restrict the impact of interventions by missing out high-prevalence populations in areas which have an overall low prevalence [49]. For example, whilst overall HIV prevalence in the City of Cape Town is lower than other districts, there are still certain subpopulations within, who are at extremely high risk. Research shows that interventions which intensify efforts in high prevalence areas whilst maintain a basic package in all locations proved most cost-effective [49]. Understanding local transmission dynamics may help to tailor the response, optimise resources and achieve maximum impact [50].

It is worth noting that interventions, as well as implementation strategies often do not discriminate between those at high risk and at lower risks. While models investigating the effect of population wide combination HIV prevention interventions have suggested that such strategies could generate reductions in HIV prevalence across the population [51], the impact of interventions depends on their ability to reach those at highest risk of acquisition [52]. Interventions which prioritise high risk individuals and localities with high prevalence reflect greater cost–benefit ratios and potential health impact of HIV prevention interventions [53]. Striking the balance between wide population coverage and high-risk targeting represents one of the key implementation challenges [49]. Whilst the intervention that is the subject of this study does provide adequate general population coverage, focussing on reaching those who are no longer in school might optimise the impact of the intervention in reducing HIV transmission. Strategies that identify the highest risk individuals within the general population, such as community-based risk assessments, may help provide a more focussed intervention while increasing coverage for the general population and thus, could be considered going forward. Therefore, identifying key risk factors, as we have done in this study, is an important step to specifically tailor interventions to meet the needs and ultimately reduce HIV transmission within that community [53].

The limitations of this study include first the cross-sectional study design limits our ability to understand the temporal link between participants’ HIV risk and intervention exposure. This is further compounded by the timing of the study (predominantly in the second year of intervention implementation), when the intervention might not have had enough time to impact AGYW’s risk behaviours. The validity of using self-reported behavioral measures as indicators of HIV risk is undermined by potential social desirability bias and recall bias. Also, such measures do not capture the sexual network factors that affect HIV incidence. Participants’ intervention exposure status was difficult to ascertain due to the absence of branding of some of the intervention components, and therefore the validity of participants’ reports of participation in the intervention is unknown. Intervention exposure is defined as having one or more exposures to branded components of HIV prevention intervention. Thus, exposure may range from a single attendance of one component to regular attendance or more than one intervention components, and we have not disaggregated the various degrees of intervention exposure. Furthermore, it was not possible to enquire about dosage or quality of the intervention due to the nature of the implementation strategy/approach, which was dependent on the implementers and on targets set by the funder, and that our role was limited to evaluating the intervention outcomes. We also did not perform sensitivity tests account for the excluded cases. However, this was a very large survey and therefore the excluded cases have no significant impact on the current findings. Lastly, the HERStory Study was initially powered to detect a 33% reduction in HIV incidence from a baseline of 3% or 4%, over two years. However, due to logistical challenges, we were not able to recruit the required ample size of 14,000 AGYW for the 1^st^ survey, which would have allowed 80% power (and 5% type 1 error rate) to detect HIV incidence in the second survey that was planned to be conducted after two years from the survey. Despite these limitations, this study provided useful insights on who is exposed to HIV intervention programs and whether the most at-risk AGYW are being exposed to prevention interventions.

## Conclusions

This study provides insights into who is exposed to combination HIV interventions, and whether the most at-risk AGYW are being reached by such programs. Compared to AGYW who reported fewer HIV risk behaviours and experiences, AGYW reporting a greater number were equally likely to be exposed to and participate in the combination HIV prevention intervention. This highlights that the targeted intervention reached the vulnerable and in-need population of AGYW. Whilst we found relatively high levels of intervention exposure among school-going AGYW, more effort is needed to tailor such interventions to reach AGYW who are out of the education system. The barriers to access and uptake of such interventions among this group should be examined to guide the development of approaches to overcome such barriers. Further studies could explore the factors that influence AGYW’s access to, and coverage by such programs.

## Data Availability

The dataset used for the current study is available from the corresponding author on reasonable request.

## Appendix

See Tables 4 and 5.

**Table 4:** Table of variables used

Variable	Description and coding	
Demographic characteristics		
District	District AGYW is from as answered in survey	
Age in years	Calculated from AGYWs answered date of birth	
Level of education	Split into 3 categories, ‘In School’, ‘completed Grade 12’ or “dropped out before completing Grade 12”	
School absenteeism	Of those in school, those who reported being absent more than 1–3 days a week considered ‘Regularly Absent’	
Exposure to interventions		
Intervention exposed	If participant answered ‘Yes’ to having ever participated or presently participating in Woman of Worth programme, or Soul Buddyz Clubs, or Rise Clubs, or homework support programmes, or sessions in which Keeping Girls in School flipchart was used, or had received a cash incentive from Woman of Worth	
*Sexual experiences and behaviours (i.e. HIV risk factors)		
Age at sexual debut	Those who answered 14 or younger were considered as having an ^**§**^ ‘early sexually debut’. Those who were 15 and above or who had never had sex were not considered at risk	
Frequency of condom use with casual partner in the past 3 months	‘Always’ was considered consistent condom use. ‘Most of the time’, ‘Sometimes’ and ‘Never’ considered inconsistent. Those who had never had sex were not considered at risk	
Frequency of condom use with main partner in the past 3 months	As above	
^§^Number of Sexual Partners in past 3 months	≥ 2 was considered multiple sexual partners and thus a risk factor. ≤ 1 were considered not at risk. who had never had sex were not considered at risk	
^§^Sexual partner more than 5 years older in the past year	‘Yes’ was considered a risk factor. ‘No’ was considered not at risk. Those who had never had sex were not considered at risk	
Sexual partner living with HIV in the past year	‘Yes’ was considered a risk factor. ‘No’, was not considered as a risk factor. Those who had never had sex were not considered at risk	
Transactional sex	Selecting any item or describing another item was considered as having transactional sex. ‘I have not done this’ was considered not at risk. Those who had never had sex were not considered at risk	
Forced to have sex	‘Once’ and ‘More than once’ considered as risk factors. ‘Never’ considered not at risk. Those who had never had sex were not considered at risk	
Sex under influence of alcohol or drugs	‘Yes’ and ‘Yes, more than once’ considered as risk factors. ‘No’ classed as no risk. Those who had never had sex were not considered at risk	
*HIV knowledge and beliefs		
Witchcraft can cause HIV	Agree/disagree	
Man with HIV can be cured by having sex with a virgin	Agree/disagree	
^§^Circumcised males cannot get HIV	Agree/disagree	
Possible to get HIV through anal sex	Agree/disagree	
HIV status		
Accessed HIV Test	‘Yes’ or ‘No’. No was considered as low access	
HIV Status	Measured via 4^th^ Generation ELISA screening tests. Classified as HIV negative if seronegative on both tests. If seropositive on both tests, and on further confirmatory Western Blot test, then participant was classified as living with HIV	

*Subscale items included in the HIV risk index scale

^**§**^Additional HIV risk factors included in the HIV risk index scale

**Table 5:** Description of the combination HIV prevention intervention components

Name	Description	Intervention components
Soul-Buddyz Club	An in-school peer-education/ youth club model in primary schools for children struggling academically, affected by HIV or with signs of neglect Clubs were facilitated by educators, who attended annual training, and used age-appropriate material	Biomedical Linkage and referral to health and other services Behavioural SRH education and peer support Structural Promote access to grants Promote an environment for ongoing learning Social cohesion
Keeping Girls In-school	A high school-based intervention for adolescent girls at risk of dropping out of school including those affected by HIV, with caregiving responsibilities or with signs of neglect. It aimed to identify and support female learners who were at risk of dropping out of school prematurely. It comprised of a peer education programme facilitated by Peer Group Trainers or Health Educators	Biomedical HIV testing; TB, STI and GBV screening; Linkage and referral to services Behavioural SRH education; peer support; home visits to encourage school attendance Structural Career guidance; homework support; Promote an environment for ongoing learning
RISE Clubs (In-school)	The Rise clubs are constituted by 15–20 young women from a school, who meet regularly to discuss issues that affect them. The clubs also linked young women to career guidance through career jamborees and homework support. The curriculum is contained in Rise magazines	Biomedical Linkage and referral to health services including HCT, PMTCTE, ART, SRH Behavioural SRH education; caregiving support; peer support; build self-efficacy and resilience Structural Social cohesion Community activism Career guidance
RISE Clubs (Out-of- school)	The clubs are constituted by 15–20 young women from a community, who meet regularly to discuss issues that affect them. The clubs also linked young women to educational and economic opportunities and local microenterprise development organisations	Biomedical Linkage and referral to health services including HTS, PMTCT, ART, SRH services Behavioural SRH education; caregiving support; peer support; build self-efficacy and resilience Structural Social cohesion Economic strengthening Community activism
Health and welfare jamborees	These events were held in school or community venues and brought health, social and other services to communities to facilitate access for AGYW and their communities	Biomedical HTS; TB, STI and GBV screening; linkage and referral to health services Behavioural SRH education Structural Career opportunities; social grants; birth registrations
Community dialogues	Targeted at men and women 14 years of age and above living in the areas of the AGYW intervention. Trained facilitators used promotional materials to guide dialogues in school or community venues. They promoted gender equity, prosocial male norms, and the uptake of men’s SRH services	Biomedical Linkage and referral to health services Behavioural SRH education Structural GBV prevention

*TB* tuberculosis; *STI* sexually transmitted infections; *GBV* gender-based violence; *HTS* HIV testing services; *PMTCT* prevention of mother-to-child transmission; *ART* antiretroviral therapy; *SRH* sexual and reproductive health
